# Acoustic, Mechanical, and Thermal Characterization of Polyvinyl Acetate (PVA)-Based Wood Composites Reinforced with Beech and Oak Wood Fibers

**DOI:** 10.3390/polym17020142

**Published:** 2025-01-08

**Authors:** Youssef Cherradi, Camelia Cerbu, Ioan Calin Rosca, Adnane Seman, Hamid El Qarnia, Ahmed Dimokrati, Mustafa Benyoucef

**Affiliations:** 1 LAMIGEP Laboratory, Moroccan School of Engineering Sciences, Marrakech 40000, Morocco; y.cherradi@emsi.ma; 2 Department of Mechanical Engineering, Faculty of Mechanical Engineering, Transilvania University of Brasov, B-dul Eroilor 29, 500036 Brasov, Romania; icrosca@unitbv.ro; 3 CRMEF Laboratory, Centre for Education Training and Formation, Marrakech 40000, Morocco; seman.adnane@gmail.com; 4 Fluid Mechanics and Energetics Laboratory (affiliate to CNRST, URAC 27), Department of Physics, Semlalia Faculty of Sciences, Cadi Ayyad University, Marrakech 40000, Morocco; elqarnia@uca.ac.ma; 5 Institut Jean Lamour, Université de Lorraine, Campus ARTEM, Allée André Guinier, F-54011 Nancy, France; ahmed.dimokrati@univ-lorraine.fr; 6 Research Laboratory for Sustainable Development and Health, Department of Applied Physics, Faculty of Sciences and Technics, Cadi Ayyad University, Marrakesh 40000, Morocco

**Keywords:** wood composites, acoustic properties, thermal properties, mechanical properties

## Abstract

Considering the growing need for developing ecological materials, this study investigates the acoustic, mechanical, and thermal properties of wood composites reinforced with beech or oak wood fibres. Scanning electron microscopy (SEM) revealed a complex network of interconnected pores within the composite materials, with varying pore sizes contributing to the material’s overall properties. Acoustic characterization was conducted using a two-microphone impedance tube. The results revealed that the fibre size significantly impacts the sound absorption coefficient, demonstrating that the highest sound absorption coefficient of 0.96 corresponds to the composites reinforced with oak wood fibres with a size of 2 mm in the low-frequency range of 1000–2500 Hz. Mechanical testing revealed a significant reduction in compressive strength as fibre size increased from 0.4 mm to 2 mm, correlating with the observed changes in sound absorption and thermal properties. Thermal analysis indicated thermal conductivity (λ) values ranging from 0.14 to 0.2 W/m·K, with a notable increase in conductivity as fibre size decreased. It was shown that composites reinforced with beech or oak wood fibres with a size of 2 mm are recommendable for insulation materials due to the lowest thermal conductivity of 0.14 W/(m·K). Oak wood composites with a fibre size of 0.4 mm recorded the highest heat capacity, which is 54.4% higher than the one corresponding to the composites reinforced with the largest fibres. The results regarding heat diffusion rates are also reported. The findings about the effects of fibre size and pores on thermal, acoustic and mechanical properties provide valuable insights for designing sustainable materials, offering potential applications in industries where balanced performance across multiple properties is required.

## 1. Introduction

Composite materials based on wood fibres or vegetable textile fibres have received significant attention due to their unique combination of renewable resources [1,2,3,4]. The increasing global focus on sustainable materials has driven strong interest in innovative composites that combine the benefits of synthetic polymers with renewable resources. One such promising approach is the integration of polyvinyl acetate (PVA) with wood sawdust, resulting a composite material with potential applications across various fields. PVA is a widely used synthetic polymer that offers several advantages, including strong adhesive properties and biodegradability. It demonstrates particularly high efficiency in bonding with porous materials like wood. PVA has proven its versatility across numerous applications over the past decade [5,6]. In line with the growing emphasis on sustainable practices, researchers have turned their attention to incorporating PVA with renewable materials [7,8]. Wood sawdust, a byproduct of wood processing industries, presents itself as a sustainable alternative, contributing to the circular economy through waste repurposing [9,10]. The conversion of wood sawdust aligns with multiple principles of green chemistry and green engineering, emphasizing renewable processes over resource-depleting alternatives [11,12]. These ideals are supported by the industry’s dedication to employing renewable resources, such as wood, and maintaining this strategy is vital for a sustainable future. Studying such composite materials involves investigation of a wide range of properties. Thermal properties, such as thermal conductivity and heat capacity, are crucial for understanding material behaviour in scenarios where temperature is a critical parameter. Acoustic properties, including the sound absorption coefficient, the frequency response, and impedance characteristics, play pivotal roles in various applications ranging from construction to soundproofing.

The combination of PVA with wood sawdust emerges as a highly promising solution that not only preserves the desirable properties of PVA, but also reduces environmental impact through the use of a renewable resource. Practical applications may range from eco-friendly construction materials to acoustic panels designed for noise control.

The state of the art of the literature regarding the characterization of the wood-based composites shows that there are other commercially relevant thermoplastic polymers like polypropylene (PP), polyethylene (PE) and polyvinyl chloride (PVC), which offer more advantages for such composites [13,14,15]. It was proved that the composites based on PP, reinforced with natural fibres (especially the ones reinforced with hemp and kenaf fibres) or with wood fibres, have better performance in terms of the specific modulus compared to the PP-based composites reinforced with short glass fibres [13]. Although mechanical strength, including impact resistance, is reduced, wood fibre-reinforced PP-based composites are recommended for their reduced abrasiveness in manufacturing and economic advantages compared to conventional glass or carbon fibre-reinforced composites [13]. Looking for other wood composites with potential for industrial applications, the literature has also revealed that the thermal and mechanical properties of the PE-based wood composites depend on the wood fibre content and wood fibre type, and these properties can be improved by adding coupling agents in their composition [14]. PVC/wood composites represent another category worldwide used especially in the window/door profile industry, with undeniable economic potential for the industry. Recent reviews [15] highlight that hybridization of the wood fibres with glass fibres or mica leads to the improvement of the mechanical properties of PVC-based composites. Compared to PVA-based composites reinforced with wood fibres, those based on polyolefins or PVC require high-performance manufacturing technology, with a greater investment in injection equipment. Acoustic and thermal properties of several wood waste-based materials have been thoroughly studied over the past decade [16,17,18,19]. These studies highlight that choosing natural, biodegradable glue is preferable for minimizing the environmental impact of these kinds of panels. This approach maintains thermal properties while simultaneously enhancing acoustic properties. The effects of different fibre sizes, bulk densities, thicknesses, and air gaps have been analysed in samples made of glued wood chips [7]. Increasing the thickness of these samples introduced additional resonance peaks, shifting the original peak to lower frequencies.

This paper presents a comprehensive analysis of the thermal, acoustic, mechanical, and moisture properties of PVA wood sawdust composites. The study investigates the use of two distinct types of wood fibres, derived from beech and oak wood, as reinforcements in composite materials. The choice of beech and oak is due to their abundance in the Transylvania region of Romania, offering a sustainable and locally-sourced solution. Composite samples were produced using different wood fibres of different sizes. The analysis begins by examining the acoustic absorption properties of the composites, a critical property for applications in sound insulation and absorption. Additionally, the moisture content of the composites was analysed to evaluate material behaviour and performance under various environmental conditions by quantifying the composite water content. The mechanical integrity of the resulting composites was evaluated by determining their compressive strength, which reflects their ability to withstand loads that compress the material. Finally, the thermal characteristics of these wood-based composites were assessed using a hot-disk device. This device enabled the measurement of thermal conductivity, heat capacity, and thermal diffusivity, providing valuable insights into the thermal performance of the composites. Through this multi-faceted analysis of mechanical, thermal, acoustic and moisture-related properties, this study aims to offer valuable insights into the performance of wood-based composites and their potential applications in various fields.

## 2. Materials and Methods

### 2.1. Materials

Composite samples tested in this research were fabricated using two types of wood fibres: beech and oak. The fibres, sourced as sawdust of varying sizes, were selected due to the common presence of beech (Fagus sylvatica) and oak (Quercus robur) trees in the Transylvania region of Romania. Beech trees are known for their smooth grey bark and dense wood, which is highly valued in furniture and flooring industry fields. On the other hand, oak trees are recognized for their sturdy, mechanical strength and distinctive growth ring patterns, making them popular for furniture, barrels, and flooring.

The matrix material used was Super Sticky adhesive for wood, manufactured by SC EVOLOR SRL (Mihesti, Romania), which is based on PVA in aqueous dispersion. This PVA-based adhesive has a density of 1.0–1.3 g/cm^3^, a Brookfield viscosity of 5000 mPa.s, and shear strength of 103–108 daN/cm^2^ [20]. Thermoplastic polymer resin, known as PVA, has a molecular weight of 90,000 g/mol and a melting point of 200 °C. PVA is particularly effective for bonding porous materials such as paper, textiles, and wood. One of the significant advantages of using PVA is its ability to dry at room temperature, thus avoiding the need for energy-intensive high-temperature drying processes. This property makes PVA an efficient and environmentally friendly choice for the composite manufacturing process.

### 2.2. Samples Manufacturing

**Figure 1:**
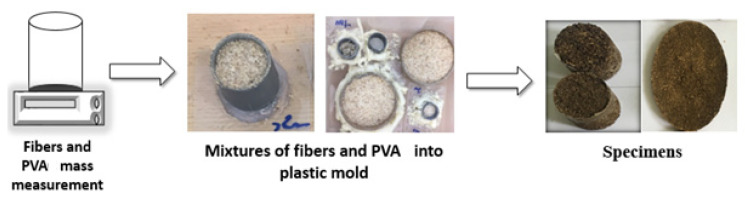
Steps involved in the manufacturing of the tested samples.

The sawdust was sieved and classified into three distinct size classes, as shown in Table 1. The manufacturing procedures for the samples were similar to those described in reference [21]. The wood fibres were weighed using a balance, and different fibre amounts [21] were used to produce samples with varying densities. The sawdust and PVA combinations were mixed in various proportions within a cup. The blend was then placed into a plastic tube mold (see Figure 1). The samples underwent a 48-h drying process at room temperature (25 °C). Once completely dry, the samples were weighed, and their weight in the dry state was used to calculate the apparent density.

### 2.3. Experimental Methods

#### 2.3.1. Analysis with Scanning Electron Microscope (SEM)

The morphological analysis of the samples was performed using a HIROX SH4000M Scanning Electron Microscope (SEM) manufactured by HIROX Co., Ltd. (Tokyo, Japan), which is operated at 10 kV. This technique enabled a comprehensive examination of the structural features and surface characteristics of the material, providing detailed insights into its microstructure in order to find correlations between the particularities of the structure on the one hand and the thermal and acoustic properties on the other hand.

To characterize the samples, a systematic procedure was followed. First, samples were prepared from cut surfaces that underwent careful manual handling to ensure clean and precise cuts. These surfaces were then mounted on SEM stubs using double-sided carbon tape to ensure stability during imaging. During SEM imaging, the focus was on capturing high-resolution images to observe the distribution and orientation of the fibres within the composite structure. Qualitative observations were made regarding the interfacial bonding between the wood particles and the PVA matrix, as well as to identify any surface irregularities or structural defects.

#### 2.3.2. Water Absorption Test

The moisture content, also known as water content, indicates the proportion of water absorbed in the wood composite and is commonly expressed as a percentage with respect to the mass of oven-dried wood composite. The standard reference ASTM D4442-20 [22] was used to determine the moisture content of raw materials with dimensions of 25 × 25 × 25 mm^3^. This involved measuring the sample’s weight before and after drying in an oven at a specific temperature of 103 ± 2 °C for a duration of 24 h. The drying period, in this case 24 h, allows sufficient time for the heat to uniformly remove moisture from the wood samples, ensuring accurate and reproducible moisture content measurements.

The water absorption test is conducted by complete immersion of all samples in water and weighing them periodically until the content of the water absorbed remains constant (which is called saturation). The aim of this test is to measure the water content at saturation.

#### 2.3.3. Acoustic Tests

**Figure 2:**
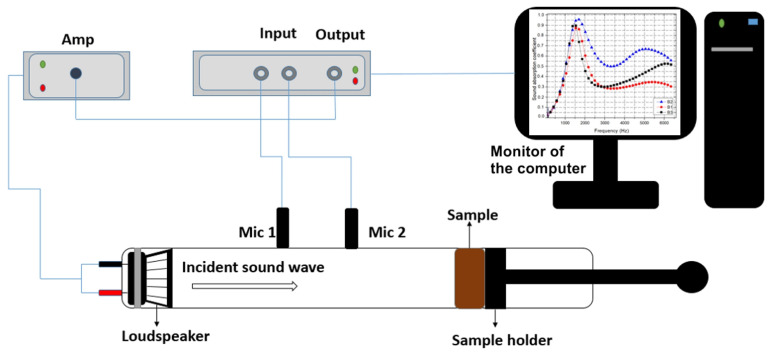
Sound absorption experimental setup (Legend: Mic 1, Mic 2—microphones; Amp—amplifier).

In compliance with ASTM E1050 [23], the two-microphone impedance tube, namely the Type 4206-A Brüel & Kjær, manufactured by Hottinger Brüel & Kjær GmbH (Virum, Denmark) was used to evaluate the acoustic absorption characteristics. A 20 °C ambient temperature, relative humidity of 80%, air density of 1.202 kg/m^3^ and sound speed of 500 m/s were all used in the measurements. PULSE LabShop software, version 12.0.0, was used to process the collected data. The experimental setup is shown in Figure 2.

The main objective of the acoustic tests was to measure the sound absorption coefficients for the wood composites involved, for different frequency ranges, in order to characterize them for acoustic performance as insulation materials. The effects of the length of the air gap between the acoustic sample and the rigid back wall on the sound absorption coefficients is also investigated in this research.

To determine the sound absorption coefficient across a frequency range of 500–6400 Hz, three samples of each composite type (see Table 1) were analysed, for a total of 18 samples, each with a diameter of 29 mm. Additionally, the sound absorption coefficient in the lower frequency range of 100–1800 Hz was measured using six samples, each with a diameter of 100 mm.

Using a loudspeaker, this technique generates a sound signal at one end of the impedance tube. By measuring the transfer function between microphones 1 and 2, the complex acoustic transfer function H_21_ is determined (see Figure 2). The material’s reflection coefficient R is then calculated by applying Equation (1):

(1) $$R=\frac{H_{21}-e^{-jks}}{e^{jks}-H_{21}}e^{2jk\left(l+s\right)},$$

where *s* is the distance between the first and second microphones; *l* is the distance between the specimen and the second microphone; *k* = 2*πω*/*c* is the wave number; *c* is the speed of sound in the air; *ω* is the frequency, and *j*^2^ = −1. Following Equation (2), the reflection coefficient can be calculated to determine the absorption coefficient *α* of the material.

(2) $$\alpha =1-{\left|R\right|}^{2}$$

Samples with greater average values for the sound absorption coefficient were subjected to measurements using air-gap widths of 10 mm and 20 mm.

#### 2.3.4. Compression Test

The capacity of a material or a structure to bear loads that tend to compress it is known as its compressive strength. This crucial mechanical characteristic can be used to identify the compressive strength and modulus of elasticity, which are essential properties in the development of useful acoustic and/or sound insulation panels. To assess the compressive strength of the six samples with dimensions of 25 × 25 × 25 mm^3^, a universal testing machine of type KM-50KN was employed. The test was conducted at the Faculty of Sciences and Technics (Marrakesh, Morocco), with a crosshead speed rate of 0.5 mm/min, in accordance with ASTM D1037 standard [24].

#### 2.3.5. Thermal Tests

In this study, the TPS 1500 hot-disk method was used to determine the thermal properties of the composite material: thermal conductivity, heat capacity and thermal diffusivity. These properties are required to characterize and recommend the materials involved in this research for the manufacturing of the thermal insulation panels. All thermal tests were performed at the Faculty of Sciences and Technics (Marrakesh, Morocco). During the measurement, the Hot Disk^®^ sensor, manufactured by Hot Disk AB (Göteborg, Sweden), is positioned between two samples of the same material with dimensions of 2.5 cm × 2.5 cm. The device then produces heat and functions as a dynamic temperature sensor. Using this transient plane source approach, the device is able to determine the different thermal properties mentioned above [25].

## 3. Results and Discussions

### 3.1. Results Obtained by Scanning Electron Microscope

**Figure 3:**
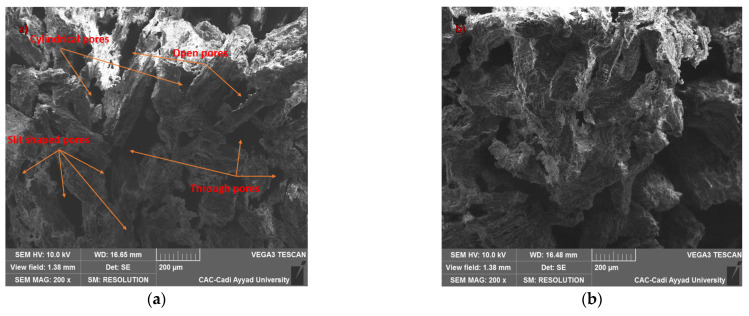
SEM image showing the microstructure of the composite specimens reinforced with (**a**) oak wood fibres; (**b**) beech wood fibres.

**Figure 4:**
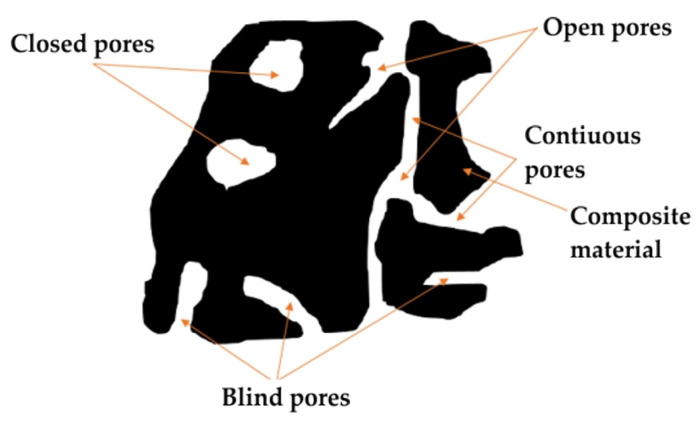
Illustrative schematic depicting various pore types.

Using SEM, we investigated the surface microstructure and morphology of the samples, with a specific focus on visually analysing pore sizes and their spatial distribution, both of which are main characteristics influencing the acoustic and thermal properties of the material. The microscopy images presented in Figure 3 illustrate the presence of pores of various sizes and shapes in the samples. The specimens generally exhibit a high level of porosity, featuring several open pores, including channel-like, slit-shaped, and cylindrical pores. Figure 4 shows a schematic illustration of the pore typology encountered in the different samples. These observations provide insights into the microstructure of the material, which is crucial for understanding its properties and behaviour. Indeed, the number and size of pores have been shown to affect thermal conductivity [26]. Thermal conductivity was found to linearly decrease as porosity increased, regardless of the spatial distribution of the pores [27]. On the other hand, the open pore structure is one of the key elements for effective sound absorption. Both intra-particle and inter-particle pores contribute to sound absorption, but the contrast in permeability between these two networks suggests that the acoustical flow is predominantly governed by the inter-particle pores.

### 3.2. Moisture Content

**Figure 5:**
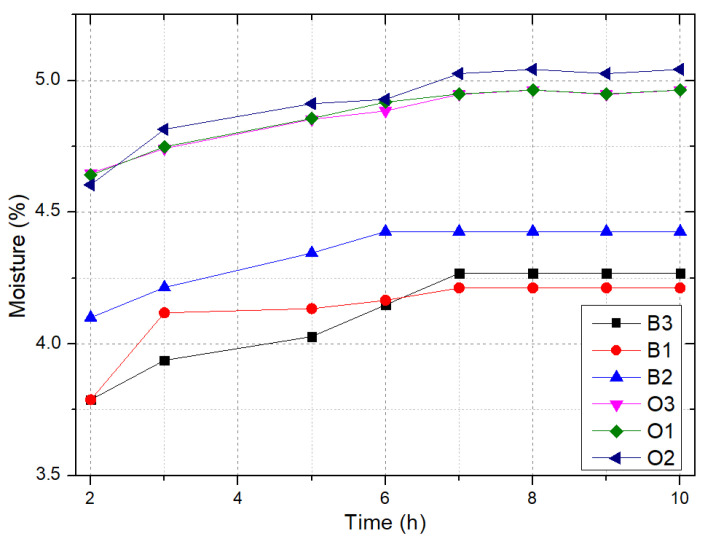
Moisture content versus time (each point represents the average of three distinct measurements).

The moisture content during testing has a notable impact on the physical and mechanical properties of building panels. In the case of samples reinforced with oak wood fibres, it is clear that the moisture content is almost insensitive to the fibre size, while for beech samples, the moisture content is slightly influenced by the fibre size. This is particularly true for sample B2, for which the moisture content is relatively higher than in the case of the B1 and B3 samples. Results are summarized in Table 2 for all samples. Moreover, there was a slight increase in moisture content from beech to oak, as shown in Figure 5.

In terms of protecting the fibres from water absorption, PVA plays a crucial role. It adheres to the surfaces of the fibres, effectively forming a protective layer that blocks a significant amount of hydroxyl groups. This, in turn, reduces the contact between water and natural fibres, resulting in enhanced water resistance. A similar outcome was shown in [28]. The moisture absorbed by the samples primarily occupies the pore volumes.

The moisture content is an indicator of how firmly the matrix is adhering to the wood fibres (Table 2). Considering these data, we can observe that the highest moisture content of 5.04%, after 10 h of immersion in water, was obtained for oak wood composites with fibre of 2 mm size, which is 13.77% higher than that recorded for beech wood composites with the same fibre size.

### 3.3. Sound Absorption Properties

#### 3.3.1. Effects of the Wood Fibre Size

**Figure 6:**
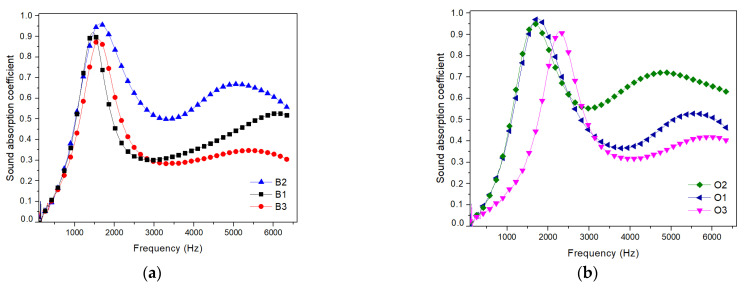
Effects of the fibre size on the sound absorption coefficient for the composite materials reinforced with (**a**) beech wood fibres; (**b**) oak wood fibres.

The sound absorption spectra at normal incidence in the frequency range of 500 to 6400 Hz for beech and oak-based composites are shown in Figure 6. The influence of different fibre sizes on the acoustic properties of the wood composites was investigated while maintaining the same thickness (20 mm) of the samples. For beech samples and for frequencies between 1000 and 2000 Hz, the peak is observed to occur at slightly increasing frequencies around 1500 Hz, as fibre size increases. In other words, larger fibres produce peaks at larger frequencies. The value of the absorption coefficient at these frequencies is the highest for the B2 sample (beach wood fibre of 2 mm size), reaching a value of 0.96, which is 9.09% higher than the one obtained in the case of the beech wood fibres with 1 mm length (B1 samples).

A steep drop is observed for intermediate frequencies between 2000 Hz and 5000 Hz, with values of 0.3, 0.29, and 0.5 for B3 samples, B1 samples and B2 samples, respectively.

For a higher frequency range (above 5000 Hz), the shift in the peak frequencies for different fibre sizes are more pronounced, yet the order is interestingly reversed, i.e., large fibres produce peaks at lower frequencies. Overall, the B2 sample exhibits the largest sound absorption coefficient in the different examined frequency domains.

For oak samples, and for the low-frequency range between 1000 Hz and 2500 Hz, the peaks are observed at lower frequencies for larger fibre sizes. For the O2 sample, the peak value of 0.95 occurs at around 1700 Hz, while for the O3 sample, the peak value of 0.91 occurs at around 2300 Hz. The highest value of 0.96 corresponds to the O1 sample. These values are slightly higher than in the case of beech samples.

For intermediate frequencies between 2000 Hz and 5000 Hz, the drop in the sound absorption coefficient occurs at lower frequencies for larger fibre sizes in the same manner as in the low-frequency range, with values ranging between 0.55 and 0.31 for the O2 and O3 samples, respectively. The same behaviour is observed for higher frequencies, with values ranging between 0.72 and 0.42 for the O2 and O3 samples, respectively.

For a clear comparison, results recorded for the different composite materials tested are summarized in Table 3. It can be observed that the sound absorption coefficient has the highest value of 0.96, which is the same for both beech and oak wood composites with fibres of 2 mm size, for the low-frequency range of 1000–2500 Hz (Table 3). This value is 9.09% higher than the lowest sound absorption coefficient, which was recorded for beech wood composites with fibres of 1 mm size.

In summary, the samples reinforced with oak wood fibres show a higher sound absorption coefficient in different frequency ranges in comparison to the samples reinforced with beech wood fibres. Moreover, the sound absorption coefficient increases monotonically with increasing fibre size in oak samples, while it shows no monotonic behaviour in the beech samples. Further analysis over a wider range of fibre sizes seems necessary for the beech wood samples to obtain more conclusive results.

It is worth mentioning that these results are qualitatively in good agreement with the moisture content analysis performed in the previous section, where it was shown that the oak-based samples exhibit higher porosity than the beech-based samples. Indeed, porosity seems to play a crucial role in the acoustic absorption properties of the composite material.

The interaction between the propagating sound wave and the pore network generally results in two types of losses: viscous losses and thermal losses. Viscous losses occur in the boundary layer of air surrounding the pores due to friction. These are known to primarily occur at low frequencies. Thermal losses, on the other hand, result from the compressions and rarefactions of sound waves, which cause slight temperature variations in the medium, converting acoustic energy into thermal energy. Thermal losses become more significant at higher frequencies. Higher porosity is thus associated with higher sound absorption performance [29,30,31].

#### 3.3.2. Air Gap Effect

**Figure 7:**
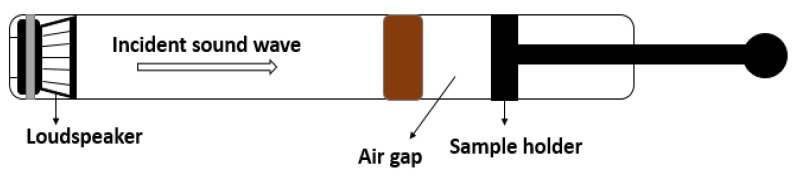
Schematic of the experimental setup used for investigating the air gap effect.

To improve the effectiveness of the acoustic absorbers, particularly for thin materials, the use of an air gap to influence the sound absorption coefficient is a well-established technique. The experimental setup involved introducing an air gap between the tested sample and the rigid back wall (Figure 7). Notably, this configuration led to an improvement in sound absorption performance, especially at lower frequencies. The spectroscopic analysis revealed a shift of the initial absorption peaks at lower frequencies, accompanied by a decrease in the sound absorption coefficient values. This beneficial effect provides a means to enhance the sound absorption at low frequencies without the need to increase the thickness of the absorber.

**Figure 8:**
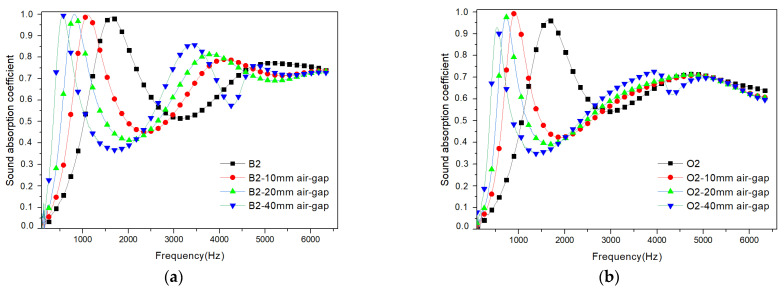
Variations of sound absorption coefficient recorded for different air gap sizes, in composite materials reinforced with different types of wood fibres: (**a**) beech wood fibres; (**b**) oak wood fibres.

Both oak- and beech-based samples with a fibre size of 2 mm exhibited higher sound absorption coefficients in the frequency range between 500 Hz and 6400 Hz (Figure 8). Consequently, these samples were chosen to explore and evaluate the influence of an air gap on their sound absorption characteristics. As expected, in the frequency range between 500 Hz and 2500 Hz, there was a shift of the peaks towards lower frequencies, along with a reduction in the full width at half maximum as the air gap increased, which contrasts with results found in the literature [32,33]. The beech-based samples showed a very slight increase in the peak value of the sound absorption coefficient. However, in the case of the oak-based samples, the peak decreased. For the frequency range of 2500 Hz to 6400 Hz, all the samples exhibited practically the same behaviour, indicating that the air gap had no effect on the middle to high frequencies.

In order to investigate the acoustic properties of the panels in the 100 Hz to 1600 Hz range, we used samples with a diameter of 100 mm and a thickness of 20 mm, reinforced with oak and beech wood fibres with a size of 2 mm. In building acoustics, low-frequency sounds can include rumbling noises, deep bass, or vibrations caused by external sources such as traffic, or machinery noise, ranging from 100 Hz to 1000 Hz. This frequency range is significant for speech intelligibility, as it encompasses the fundamental frequencies of human speech. Frequencies above 1000 Hz extend into the upper range of human hearing. This range is important for sound clarity, music reproduction, and overall acoustic quality in spaces such as concert halls, recording studios, or auditoriums.

**Figure 9:**
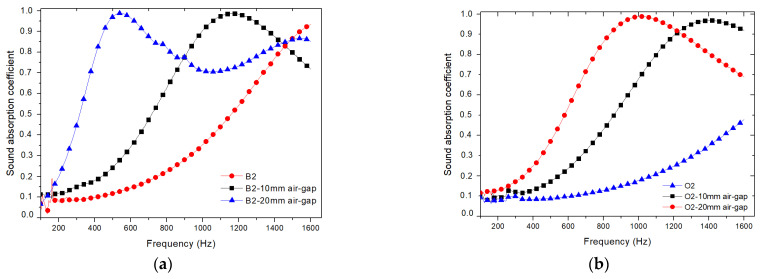
Variations of sound absorption coefficients recorded at low frequencies with different air gap lengths: (**a**) beech wood composite B2; (**b**) oak wood composite O2.

By adding an air gap, the panels’ sound absorption properties have been significantly shifted towards lower frequencies, improving their performance as building acoustic insulation. The inclusion of a 10 mm air gap for oak-based samples significantly affects the sound absorption properties, particularly in the lower frequency range (see Figure 9). The air gap acts as a resonator, facilitating better dissipation of sound energy at lower frequencies [32], thereby enhancing the overall effectiveness of the beech-based panels as building acoustic insulation [33]. Similarly, employing a 20 mm air gap for both oak- and beech-based specimens also yields notable improvements in sound absorption. The larger air volume provided by the 20 mm gap allows for enhanced attenuation of low-frequency noises, making the panels more effective in reducing rumbling sounds and vibrations commonly encountered in building environments [34]. By utilizing these specific air gap dimensions (10 mm for oak-based specimens and 20 mm for oak- and beech-based specimens), the panels are able to cover a wider range of frequencies and provide improved energy insulation.

### 3.4. Compression Properties

The processed results from the compression tests revealed variations in both elasticity modulus and compression strength. The mechanical properties of these new composite materials are significantly influenced by both arrangement of fibres and interactions between the polymer matrix and the surface of the fibres [35].

As depicted in Table 4, a significant decline in compressive strength was evident for the specimens containing wood fibres with a size of 2 mm compared with the ones containing wood fibres with a size of 0.4 mm. In the case of oak samples reinforced with fibres with a size of 0.4 mm, the compression strength measured was highest (i.e., 0.030 ± 0.007 MPa). However, for the composites reinforced with fibres having a size of 2 mm, a substantial reduction of 54.5% was observed, resulting in a compression strength of 0.014 ± 0.003 MPa. This observed trend was similarly recorded for the compressive modulus of elasticity. In the context of beech-based samples, those with fibres having a size of 0.4 mm or 1 mm exhibited comparable behaviour, while those containing wood fibres with a size of 2 mm showed a significant decrease.

The observed reduction in the compressive strength as the size of the reinforcing wood fibres increased from 0.4 mm to 2 mm is correlated with changes in sound absorption properties and thermal characteristics. This observation matches those of other researchers [20,36]. Longer fibres contribute to alterations in the arrangement of the material pores, impacting both mechanical and acoustical features. Interestingly, the beech-based composites containing wood fibres with a size of 0.4 mm and 1 mm exhibited comparable acoustic and mechanical properties, which may be attributed to the distinct roughness characteristics of beech wood compared to oak wood. Specifically, the roughness of beech fibres, in both longitudinal and radial directions of the wood, likely accounts for the similar behaviour observed for these fibre sizes, in contrast to the changes noted with larger fibre sizes [37].

### 3.5. Thermal Properties

The data highlight key differences among wood fibre reinforcements regarding thermal conductivity, diffusion, and specific heat capacity (Table 5). Wood composites based on beech or oak fibres with a size of 2 mm, coded with B2 and O2, respectively, are the most effective insulators, with thermal conductivities of 0.14 W/(m·K). Beech wood composites with a fibre size of 0.4 mm (type B3) exhibit the highest diffusion rate, of 0.28 m^2^/s, which is 7.69% or 16.67% higher than the ones recorded for 2 mm or 1 mm fibre sizes, respectively, allowing for rapid heat, whereas oak wood composites of types O1 and O3 exhibit slower diffusion (Table 5). Oak wood composites of type O3 have the highest specific heat capacity at 0.88 J/(kg·K), indicating superior heat storage, closely followed by beech wood composites of type B1. This suggests that beech wood composites of type B2 and oak wood composites of type O2 are preferable for insulation applications, considering additionally the conclusions of the research published [38] about the effects of the wood natural directions on the thermal properties. Oak wood composites of type O3 with a fibre size of 0.4 mm are the best for scenarios requiring high heat absorption, since their heat capacity is highest, and it is 54.4% higher than the one corresponding to the composites reinforced with the largest fibres.

Considering the thermal properties reported in Table 5, it can be observed that as the size of the fibres decreases, the thermal conductivity increases. This may be explained by the fact that these composite materials have fewer pores, and the size of those pores is smaller as the size of the wood fibres decreases. Inside the material, there are numerous macro-pores and micro-pores that are linked and open to the surface. The material resembles a complex network of channels with several solid and capillary structures. In porous materials, heat is propagated by three processes: thermal conductance through the solid, radiation, and convection through the continuous pores [39]. A prior study found that a larger pore size, which increases the average free path of air, can reduce heat conduction in air [40]. The efficiency of heat transmission decreases as the number of pores increases, which is explained by the fact that the heat transfer path becomes longer.

## 4. Conclusions

A comprehensive investigation into the thermal, mechanical, and acoustic properties of wood composite samples provides valuable insights into their potential applications and performance characteristics.

Acoustic analysis revealed significant findings regarding the sound absorption properties of beech- and oak-based composites. The sound absorption spectra at normal incidence, spanning frequencies from 100 Hz to 6400 Hz, showed that among the oak composites, the sample with a fibre size of 2 mm achieved the highest absorption peak, between 1000 Hz and 2000 Hz. The oak wood samples exhibited superior sound absorption coefficients with respect to those recorded for beech wood samples, ranging from 0.89 to 0.96, compared to those containing shorter wood fibres.

The highest sound absorption coefficient was 0.96% and corresponds to both wood composites tested, which were reinforced with wood fibres of 2 mm size. This highest value is 9.09% higher than that recorded for beech wood composite reinforced with wood fibres of 1 mm size.

The influence of an air gap between the wood composite sample and the rigid back wall was also investigated. Results indicate that the presence of an air gap improves the overall sound absorption coefficient, particularly at lower frequencies, by enhancing the interaction of sound waves with the porous material and increasing the effective acoustic depth.

Mechanical testing demonstrated a significant decrease in compressive strength as fibre size increased from 0.4 mm to 2 mm. This trend was contrary to the trend of the sound absorption coefficient, which is greater as the wood fibre size is greater. A tendency to a decrease in the compressive strength as the wood fibre size increases is similar to that of the tendency of the heat conductivity to decrease as the wood fibre size increases, which leads to the conclusion that wood fibres of 2 mm size should be recommendable for reinforcing such thermal insulation panels that do not require high compressive strength. The interplay between compressive strength, acoustic behaviour, and thermal properties highlights the importance of considering fibre characteristics in material design to achieve the desired mechanical and acoustic performance.

Thermal analysis showed that the biobased samples exhibited thermal conductivity values ranging from 0.14 to 0.2 W/(m·K), aligning with previous studies. Composites reinforced with both beech or oak wood fibres with a size of 2 mm are recommendable for insulation materials due to the lowest thermal conductivity, of 0.14 W/(m·K). Oak wood composites with a fibre size of 0.4 mm recorded the highest heat capacity, which is 17.3% or 54.4% higher than the one corresponding to the composites reinforced with the fibres of 1 mm size or 2 mm size, respectively. The observed increase in thermal conductivity with decreasing fibre size suggests a relationship between pore size and heat conduction efficiency. It was shown that the material’s thermal behaviour is significantly influenced by its macro-pore and micro-pore network, highlighting the need for a thorough understanding of solid conduction, radiative heat transfer, and convective processes through pores for optimizing thermal properties in diverse applications.

The highest moisture content, of 5.04% after 10 h of immersion in water, was obtained for oak wood composites with fibres of 2 mm size, which is 13.77% higher than that recorded for beech wood composites with the same fibre size.

This research provides a detailed analysis of the biobased material’s acoustic, mechanical, and thermal performances. The findings contribute to the growing body of knowledge on sustainable materials and facilitate the development of tailored designs to meet specific performance requirements. Future research in this field holds the potential for advancements in sustainable materials with optimized thermal, mechanical, and acoustic characteristics.

## Figures and Tables

**Table 1:** Properties of the wood composite materials tested.

Reinforcement Wood Fibres	Code	Properties of the Wood Fibres		Wood Fibre Content (wt.%)	Density of the Composite Materials (g/cm^3^)
		Size (mm)	Density (g/cm^3^)		
Beech wood fibres	B2	2.0	0.68	56	0.65
	B1	1.0			
	B3	0.4			
Oak wood fibres	O2	2.0	0.64		0.64
	O1	1.0			
	O3	0.4			

**Table 2:** Moisture content percentage in different wood composite samples tested.

Sample Code	B2	B1	B3	O2	O1	O3
Moisture Content (%)	4.43	4.21	4.27	5.04	4.96	4.96

**Table 3:** Sound absorption coefficients of the PVA-based wood composites tested across different frequencies and in relation to fibre sizes.

Wood Fibre Type	Code of Wood Composite Material	Low Frequencies [1000–2500] Hz	Intermediate Frequencies [2000–5000] Hz	High Frequencies [5000–7500] Hz
Oak	O2	0.96	0.55	0.72
	O1	0.95	0.36	0.53
	O3	0.92	0.32	0.42
Beech	B2	0.96	0.50	0.67
	B1	0.88	0.29	0.35
	B3	0.92	0.30	0.55

**Table 4:** Properties obtained in compression test.

Sample Code	O3	O1	O2	B3	B1	B2
Modulus of Elasticity in Compression (MPa)	0.101 ±0.013	0.044 ±0.008	0.035 ±0.015	0.064 ±0.092	0.062 ±0.010	0.020 ±0.005
Compressive Strength (MPa)	0.030 ±0.007	0.015 ±0.003	0.014 ±0.003	0.022 ±0.006	0.024 ±0.006	0.009 ±0.003

**Table 5:** Results obtained for thermal conductivity, diffusion and capacities.

Reinforcement Fibres	Coding	Thermal Conductivity (W/(m·K))	Diffusion (m²/s)	Cp (J/(kg·K))
Beech wood fibres	B2	0.14	0.26	0.53
	B1	0.18	0.24	0.76
	B3	0.18	0.28	0.65
Oak wood fibres	O2	0.14	0.26	0.57
	O1	0.17	0.22	0.75
	O3	0.20	0.22	0.88

## Data Availability

The original contributions presented in this study are included in the article. Further inquiries can be directed to the corresponding author.
